# Transporter Engineering for Enhancing Citric Acid Production in *Aspergillus niger*

**DOI:** 10.3390/jof12070472

**Published:** 2026-06-26

**Authors:** Jie Li, Mingyang Li, Yan Song, Zeyu Xu, Yue Chen, Xianli Xue, Depei Wang

**Affiliations:** 1 Key Laboratory of Industrial Microbiology & Engineering Research Center of Food Biotechnology of Ministry of Education, College of Biotechnology, Tianjin University of Science and Technology, Tianjin 300457, China; joylee6863@163.com (J.L.); limingyang@mail.tust.edu.cn (M.L.); songyansy@mail.tust.edu.cn (Y.S.); xuzy@mail.tust.edu.cn (Z.X.); yuechen@tust.edu.cn (Y.C.); 2 Key Laboratory of Industrial Fermentation Microbiology, Tianjin University of Science and Technology, Ministry of Education, Tianjin 300457, China; 3 Tianjin Engineering Research Center of Microbial Metabolism and Fermentation Process Control Technology, College of Biotechnology, Tianjin University of Science and Technology, Tianjin 300457, China

**Keywords:** transporter engineering, glucose transporter MstF, citrate exporter CexA, *Aspergillus niger*, citric acid

## Abstract

The efficient industrial production of citric acid by *A. niger* requires overcoming the limitations of substrate uptake and citrate export on the citrate synthesis efficiency. This study addresses these obstacles using a transporter engineering strategy, modifying the endogenous high-affinity glucose transporter MstF and citrate exporter CexA. The “push–pull” strategy was used to improve citric acid production by increasing glucose import and citrate export. A single overexpression of *mstF* improved citric acid production, reaching 179.35 g/L in the H7 strain. However, *cexA* high expression impaired dense mycelium pellet formation and affected the expression of key genes, resulting in reduced citric acid production. For balancing intracellular accumulation and secretion of citrate, simultaneous overexpression of *mstF* and *cexA* increased citric acid production and efficiency. In a 30 L fermenter, strain A5 achieved a citric acid titer of 185.91 g/L, a productivity of 3.21 g/h/L, and a shortened fermentation cycle. Collectively, these results provide a reference for the industrial production of citric acid and other organic acids.

## 1. Introduction

*A*. *niger* is the main industrial workhorse for global citric acid (CA) production [1]. *A*. *niger* utilizes corn starch as a raw material, producing 180–200 g/L CA within 60–80 h at 35–37 °C [2,3,4]. Whole corn flour is a nutrient-rich source that provides almost all the nutrients required for citric acid fermentation by *A*. *niger* [1]. CA production may depend on the strain, process, scale, and other operating conditions (Table 1). In industrial fermentation, increasing yield and shortening the fermentation cycle to reduce costs and increase efficiency have become the first priority. With a detailed understanding of the mechanism of CA synthesis and the development of omics, using metabolic engineering strategies to modify *A. niger* to obtain high-yielding CA-engineered strains has become a core strategy [5]. Collectively, such metabolic engineering strategies mainly include: (1) enhancing the metabolic flux of the glycolytic pathway to increase the abundance of precursors [5]; (2) blocking by-product formation [2,6]; (3) regulating NAD(H) recycling and decoupling the feedback inhibition of ATP [7]; and (4) engineering the transporter to enhance substrate import and citrate export [8]. Currently, transporter engineering is an attractive strategy for increasing metabolite production and reducing downstream extraction costs [8,9,10]. Carbon source uptake and intracellular metabolite export are two major bottlenecks in industrial microbial fermentation [9]. Both of these obstacles can be overcome by genetic manipulation to increase expression levels or transport activity [9].

Transporters that import substrates can increase the substrate absorption rates and promote the flow of substrate to the target product [14]. *A. niger* has a high and low-affinity dual glucose transport system. The high-affinity transport system was inhibited at high glucose concentrations and assisted the low-affinity transport system in a low-capacity, high-affinity manner [15,16]. Increasing the number of high-affinity glucose transporters achieved the uptake rate of low-affinity transporters [15]. However, sugar transporters are regulated by complex transcriptional regulators and have different affinities for substrates [17,18]. In *A. niger*, MstA, MstE, MstF, MstG, and MstH were identified as high-affinity glucose transporters, whereas MstC was identified as a low-affinity glucose transporter [16,19]. In *A. niger* citric acid fermentation, a low-affinity transporter was continuously expressed at high levels, while five high-affinity glucose transporters were induced for expression [20]. Increasing the expression of high-affinity glucose transporters can ensure glucose absorption rates and maintain citrate synthesis flux.

Promoting the secretion of intracellular metabolites is also an important target for metabolic engineering to enhance industrial organic acid production. Improving the secretion of organic acids eliminated cytotoxicity and the burden caused by intracellular accumulation [8]. Increasing carboxylic acid transporter expression successfully enhanced organic acid accumulation [21,22,23]. CexA was the major citrate exporter of *A. niger* and belonged to the DHA1 family [24]. Increasing *cexA* transcription increased extracellular accumulation of CA [24,25], whereas *cexA* knockout eliminated citrate secretion and decreased glycolytic flux [26,27]. CexA not only facilitated citrate secretion, but also positively promoted the CA production morphology [28]. In summary, CexA occupies an irreplaceable role in the CA fermentation of *A. niger*.

Research on the function and mechanisms involved in glucose transporters and citrate exporters in *A. niger* has been steadily increasing [16,19]. However, most research on transporters has been conducted at the laboratory scale. Transporters, as components of membranes, have not been sufficiently studied in terms of their role in filamentous morphology development, and their potential industrial significance remains largely unexplored. Glucose transporters and the citrate exporter have been demonstrated to increase citric acid production, but production levels do not reach those of the citric acid hyper-producing strain [16,29]. Therefore, it is necessary to further explore the promoting effect of transporters on the citric acid production to establish more efficient and economical metabolic control strategies and further reduce the cost of biological production.

This study used the high-producing CA strain of industrial *A. niger*, emphasizing transporters as a promising target for rapid and efficient CA production. Our findings demonstrated that individually overexpressing *mstF* augmented CA accumulation, but intracellular citrate export further limited CA production potential. Surprisingly, individually overexpressing *cexA* impaired dense mycelium pellet formation and caused intracellular metabolic disturbances, thereby leading to a decrease in CA production. To address this challenge, the simultaneous overexpression of *mstF* and *cexA* in *A. niger* enabled a dynamic equilibrium between citrate synthesis and export. Improved fermentation performance was evident, including higher CA titer and apparent sugar-to-acid yield, and shorter fermentation durations. In conclusion, our study makes a great contribution to the enhancement of industrial CA manufacturing by modifying the transporter module and serves as an excellent reference for the metabolic regulation of other organic acids.

## 2. Materials and Methods

### 2.1. Strains, Plasmids, and Media

Details of the engineered *A. niger* strains and plasmids utilized in this study are available in Tables S1 and S2. The industrial *A. niger* CGMCC 10142, as a citric acid hyper-producing strain [2], served as the parent strain. *Escherichia coli* (*E. coli*) DH5α was used for plasmid construction, amplification, and storage. *Agrobacterium tumefaciens* (*A. tumefaciens*) AGL1 was used to transfer T-DNA (including the target gene) to *A. niger* during transformation. *E. coli* and *A. tumefaciens* were cultured in a Luria broth (LB) medium. *A. niger* was cultivated in or on the indicated media. A potato-dextrose agar (PDA) medium was used for obtaining conidia [26]. The complete medium (CM) was used for the screening of transformants [19], optionally supplemented with 200 μg/mL hygromycin B, and/or 25 μg/mL bleomycin. A liquefied corn medium was used as the CA fermentation medium, as previously described [2], with an initial total sugar concentration of 180 g/L. The mineral elements of the seed medium consisted of FeSO_4_·7H_2_O 30 mg L^−1^, CaCl_2_·2H_2_O 10 mg L^−1^, MnCl_2_·4H_2_O 7 mg L^−1^, CuSO_4_·5H_2_O 0.3 mg L^−1^, MgSO_4_·7H_2_O 0.3 g L^−1^, ZnSO_4_·7H_2_O 10 mg L^−1^, and KH_2_PO_4_ 0.3 g L^−1^.

### 2.2. Plasmid Preparation

The *gas* promoter (P*gas*), *mstF* gene and terminator (*mstF*, T*mstF*), *glaA* promoter (P*glaA*), *cexA* gene and terminator (*cexA*, T*cexA*), upstream and downstream homologous arms of *ku70* (*ku70* up, *ku70* down), and upstream and downstream homologous arms of *agdA* (*agdA*up, *agdA*down) were cloned from the genome of *A. niger,* CGMCC 10142. The bleomycin (*ble*) and hygromycin B (*hyg*) selection markers were cloned from plasmids p44 and p60, respectively. The *mstF* and *cexA* expression cassettes were constructed by overlapping PCR. The plasmid p44 was digested with *Eco*RI and *Hin*dIII. The digested fragment of p44 and expression cassettes were connected using Clon Express MultiS One Step Cloning Kit (Vazyme, Nanjing, China), generating p94 and p95 (Figure S1). All primers used for plasmid construction are listed in Table S3.

### 2.3. Construction and Initial Screening of Engineered A. niger

Recombinants were constructed by *A. tumefaciens*-mediated transformation (ATMT) as described previously [2,30]. Briefly, the target gene expression cassettes in p94 or p95 were integrated into the genome of *A. niger* by ATMT. Recombinants can be easily isolated from CM medium under the stress of bleomycin and/or hygromycin B. Subsequently, transformants were verified by PCR to amplify the overexpression cassette. The *mstF* overexpression-associated transformants were further screened in the CM medium with low glucose (0.05%) and 0.2% calcium carbonate (CaCO_3_) (Tables S4 and S5). Briefly, fresh mycelium from the transformants grown on CM + hygromycin medium was transferred to CM (0.05% glucose) + 0.2% CaCO_3_ plates and incubated at 37 °C for 72 h. The strain’s CA production was assessed by comparing the ratio of the transparent halo zone to the colony diameter. A larger acid-etched cycle indicates a greater acid production potential. Subsequently, spore suspensions of the proper transformants were prepared for shake flask fermentation.

After 15 generations of the selected transformants were passaged on the CM medium, the genomic DNA was extracted to verify their genetic stability. Additionally, CA production in the shake flask fermentation was retested.

### 2.4. Shake Flask and Fermenter Cultures

The process for shake flask culture was initiated by cultivating 1 × 10^5^ spores/mL in a 50 mL medium within a 500 mL Erlenmeyer flask. Shake flask cultivation took place at 37 °C at 300 rpm for 72 h. The recombinants with the highest CA titer in the shake flask were selected for a 30 L fermenter scale-up.

The fermenter culture was initiated by inoculating 1 × 10^5^ spores/mL into a 5 L seed jar with 3 L of seed medium. The seed jar was cultured at 35 °C with a rotation speed of 300 rpm and an aeration rate of 330 L/h for 24 h. Then the culture mixture was transferred to a 30 L fermenter containing 20 L fermentation medium at 10% inoculum. The process was maintained at 35 °C with a rotation speed of 350 rpm and an aeration rate of 400 L/h for 64 h. The measurement of relevant indicators begins at 0 h post-inoculation. The entire fermentation process was operated under fully automated controlled conditions with real-time monitoring of all parameters.

Additionally, 1 × 10^5^ *A. niger* spores were inoculated into a 250 mL conical flask containing 50 mL of CM medium and then cultured at 35 °C and 200 rpm. Samples were collected at specified time points in order to determine the relative transcriptional levels of the high-affinity glucose transporter.

### 2.5. Measurement of Organic Acids, Sugar, and Biomass

The fermentation broth was centrifuged at 4 °C and 12,000 rpm for 10 min. Then the supernatant was filtered through a 0.22 μm sterile filter membrane. The contents of CA were assessed using high-performance liquid chromatography (HPLC) with an Aminex HPX-87H column (Bio-Rad, Tianjin, China) and ultraviolet detection in 5 mM H_2_SO_4_, flowing at a rate of 0.6 mL/min. In addition, the injection volume of HPLC was set as 10 μL, and the column temperature was set as 60 °C. Standard solutions of anhydrous citric acid with varying concentrations were tested under identical conditions, and a calibration curve was plotted. The concentration of citric acid in the fermentation samples was calculated by linear regression analysis based on the peak area. The content of total sugar and reducing sugar was determined using the 3,5-dinitrosalicylic acid (DNS) method according to the previous research [31]. Briefly, 1 mL of diluted supernatant and 1 mL of DNS reagent were placed in a boiling water bath for 5 min, then cooled to room temperature. Subsequently, absorbance at 540 nm (A_540_) was measured to determine reducing sugars. The total sugar in the supernatant required the addition of an equal volume of 6 mol/L HCl, which was heated in a boiling water bath for 30 min, adjusting the pH to neutral, and subsequently measured via the DNS method. Standard measurements were performed using the same procedure. For dry cell weight (DCW) analysis during fermenter cultivations, 50 mL of fermentation broth was filtered onto pre-weighted filter paper and dried at 60 °C until the weight was constant.

The calculation formulas of the fermentation results were as follows [4]:

Citric acid titer = concentration of citric acid in fermentation broth (g/L).

The apparent sugar-to-acid yield = concentration of CA/concentration of total sugar (g/g).

Citric acid productivity = concentration of CA/fermentation time (g/L/h).

### 2.6. Measurement of Relative Transcription Level

After cultivation for the indicated time, fresh mycelia of *A. niger* were washed and vacuum filtered with filter paper. The wet pellets were snap frozen in liquid N2 and powdered using a mortar and pestle (WKBIO, Tianjin, China) [30]. The total RNA was extracted with the Fungal RNA Extraction Kit (Coolaber, Beijing, China) according to the manufacturer’s instructions. The total RNA was reverse transcribed to cDNA with HiScript III RT SuperMix for qPCR (+gDNA wiper) (Vazyme, Nanjing, China). Reverse transcription-quantitative PCR (RT-qPCR) was performed to quantify the transcription level of the target gene with the ChamQ Universal SYBR qPCRMaster Mix (Vazyme, Nanjing, China). The relative transcription level was calculated via the 2^−ΔΔCt^ method [7]. The primers used for RT-qPCR in this study are listed in Table S3. The housekeeping gene *actA* in *A. niger* was used as the reference gene.

### 2.7. Statistical Analysis

Experiments and measurements in this study were performed in triplicate. All data were presented as mean values or mean ± standard deviation. Statistical analysis was performed with the Origin 2024 software package. A one-way ANOVA statistically tested all values with a Tukey’s post-hoc test at a 95% confidence interval. Asterisks indicate significant differences: * represents a *p* < 0.05; ** represents a *p* < 0.01; and *** represents a *p* < 0.001. Data are given as means ± SD, *n* = 3.

## 3. Results

### 3.1. Screening of High-Affinity Glucose Transporter

**Figure 1:**
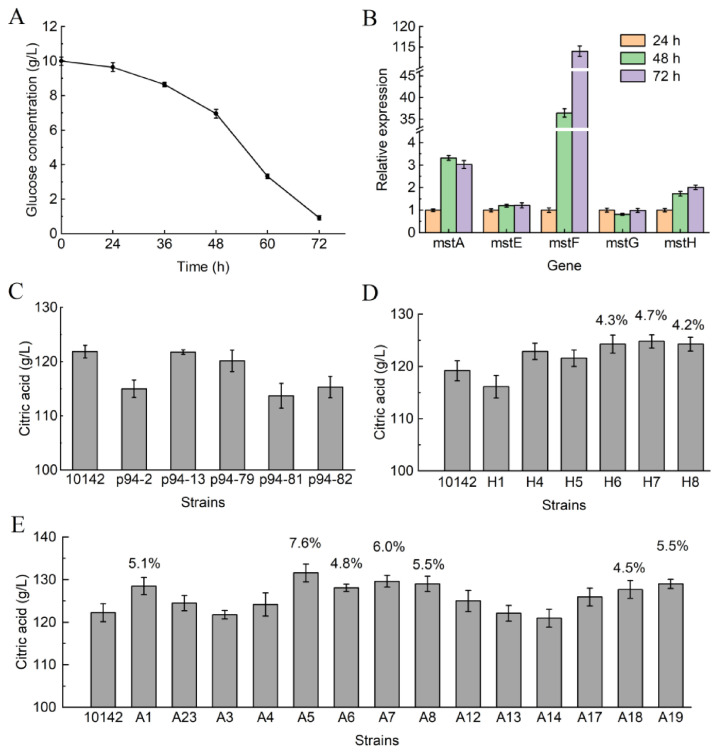
Transcriptional analysis of high-affinity transporters of CGMCC 10142 and citric acid shake flask fermentation experiments of recombinant strains. (**A**,**B**) correspond to the low-glucose CM medium experiment used to assess transporter transcription. In contrast, (**C**–**E**) correspond to citric acid shake flask fermentation screening. The glucose consumption curve (**A**) and relative expression levels of high-affinity transporters (**B**) of *A. niger* CGMCC 10142 during cell growth in the CM medium. The *cexA* overexpressing recombinants (**C**), *mstF* overexpressing recombinants (**D**), and the *cexA* and *mstF* simultaneously overexpressing recombinants (**E**) with high citric acid production were screened through citric acid shake flask fermentation in liquefied corn medium. The data presented are mean values of at least three independent assays; the error bars represent the standard deviation. Data are given as means ± SD, *n* = 3.

Glucose uptake and conversion affect the intracellular citrate synthesis rate, and the increased glucose transport significantly increased the CA production in *A. niger* [16]. So far, several key transporters in *A. niger* have been identified, including the low-affinity glucose transporter, MstC, and the high-affinity glucose transporters, MstA, MstE, MstF, MstG, and MstH. High-affinity and high-capacity exhibit clear exclusivity. Increasing the number of individual high-affinity transporters can achieve uptake rates equivalent to those of low-affinity systems [15]. To identify which high-affinity glucose transporter in CGMCC 10142 was highly induced under low glucose conditions. Therefore, we analyzed glucose consumption and the relative transcript levels of five high-affinity glucose transporters (MstA, MstE, MstF, MstG, MstH) in CGMCC 10142 grown on the low-glucose CM medium (Figure 1A,B). The *mstA*, *mstF*, and *mstH* genes were expressed upon glucose depletion (Figure 1A,B), with *mstF* showing a significant difference. Previous findings indicated that the expression of *mstF* was highly induced upon glucose depletion [19]. Ultimately, the high-affinity glucose transporter MstF was selected as the target to improve glucose uptake.

### 3.2. Construction Engineered Strains

Both vectors were introduced into *A. niger* by an ATMT, and transformants were screened. The transformants were cultivated for three generations on the CM medium supplemented with bleomycin and/or hygromycin B. Finally, 13 *mstF* overexpression transformants, five *cexA* overexpression transformants, and 14 *mstF*/*cexA* co-overexpression transformants were screened. As shown in Figures S2–S4, the random integration of *mstF* and/or *cexA* expression cassettes into transformant genomes was further confirmed by PCR. For *mstF* overexpression-associated transformants, superior transformants were screened by calculating the ratio of the clear zone diameter to colony diameter (Tables S4 and S5). Ultimately, five *cexA* overexpression transformants (Figure 1C), six *mstF* overexpression transformants (Figure 1D), and 14 *mstF*/*cexA* co-overexpression transformants (Figure 1E) were isolated through plate screening. These recombinant strains (Figure 1C–E) were then further identified for their citric acid production capacity in citric acid shake flask fermentations using liquefied corn medium. Only the CA titer of the *cexA*-overexpressing recombinant p94-13 was comparable to that of CGMCC 10142, while the titers of all other recombinant strains were lower than that of CGMCC 10142 (Figure 1C). The CA titer of the *mstF*-overexpressing recombinant H7 increased by 4.7% compared to CGMCC 10142, reaching 124.75 g/L (Figure 1D). And the CA titer of the *mstF*/*cexA* co-overexpressing recombinant A5 increased by 7.6% compared to CGMCC 10142, reaching 131.57 g/L (Figure 1E). Therefore, strains p94-13 (*cexA* overexpression), H7 (*mstF* overexpression), and A5 (*mstF*/*cexA* co-overexpression) were employed for scale-up in a 30 L fermenter. Furthermore, strains p94-13, H7, and A5 were passaged for 15 generations, and the strains’ genetic stability was confirmed by validating the *mstF* expression cassette and *cexA* expression cassette, as shown in Figure S7.

### 3.3. Inducible Overexpression of mstF Increases Glucose Consumption and CA Production

**Figure 2:**
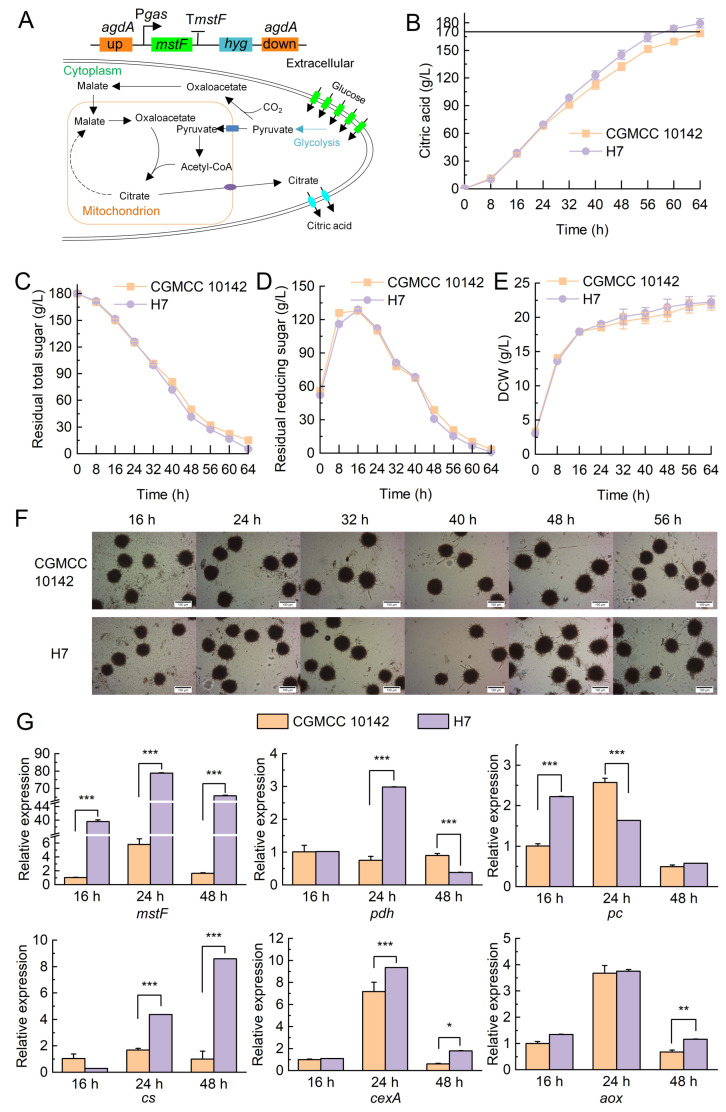
Inductive expression of *mstF* in *A. niger* using the promoter P*gas*. (**A**) Schematic diagram of *mstF* single overexpression in *A. niger*. (**B**–**E**) Citric acid, residual total sugar, residual reducing sugar, and dry cell weight (DCW) were measured every 8 h in a 30 L fermenter. (**F**) Microscopic observation of the mycelium pellet’s morphology in a 30 L fermenter. (**G**) Transcript levels of key genes involved in intracellular citrate synthesis were measured at the indicated timepoints. The data presented are mean values of at least three independent assays; the error bars represent the standard deviation. Statistical significance was estimated by one-way ANOVA followed by a post hoc Tukey’s multiple comparisons. Asterisks indicate significant differences: * *p* < 0.05; ** *p* < 0.01; *** *p* < 0.001, indicating varying degrees of significance. Data are given as means ± SD, *n* = 3.

An efficient glucose transport system was the basis for increasing the flux of citrate synthesis [20]. However, the high initial sugar concentration in citric acid fermentation inhibited the high-affinity transport system. Increasing the number of high-affinity glucose transporters improves glucose transport efficiency. Therefore, overexpression of *mstF* at the low pH-inducible promoter P*gas* enhanced citrate synthesis flux under the “push” effect (Figure 2A). In industrial *A. niger* citric acid fermentation, transcriptomic analysis revealed that the P*gas* promoter functions efficiently at a low pH (2.0), and the P*gas* strength correlates exclusively with pH [32].

In comparing the fermentation process of H7 with that of CGMCC 10142, the trend in dissolved oxygen (DO) change was nearly the same (Figure S8A). Both strains exhibited a rapid decline in dissolved oxygen to a minimum within 10 h, and remained at low levels throughout the fermentation process. The pH decreased continuously, reaching a final value of 1.5 (Figure S8B). The CA production assay revealed that the final CA titer of H7 was 6.41% higher than that of CGMCC 10142, reaching 179.35 g/L (Figure 2B). Upon calculation, the apparent sugar-to-acid yield in H7 stands at 0.99 g/g, demonstrating a productivity of 2.80 g/h/L. The monitoring of sugar consumption indicated that H7 increased the rate and efficiency of sugar utilization compared with CGMCC 10142 (Figure 2C,D). The total sugar utilization of H7 was 24.72% higher than that of CGMCC 10142, at only 5.31 g/L, indicating that MstF can improve the fermentation performance while increasing conversion efficiency. During fermentation, it can be observed that H7 formed mycelium pellets and biomass comparable with CGMCC 10142 (Figure 2E,F), illustrating that MstF has no effect on the growth or morphology of *A. niger*. Transcription analysis showed (Figure 2G) that the relative expression levels of *mstF*, *pdh*, *pc*, *cs*, *cexA*, and *aox* genes in the H7 were 21.85-, 1.65-, 1.09-, 3.54-, 1.39-, and 1.17-fold those of CGMCC 10142, respectively. Transcript levels increased significantly for *cs*, but not for *cexA*, so it is hypothesized that intracellular citrate export limited the CA production potential of H7. In conclusion, the *mstF* overexpression successfully increased the CA titer and the apparent sugar-to-acid yield.

### 3.4. Overexpression of cexA Impairs Mycelium Pellet Morphology and Reduces CA Production

**Figure 3:**
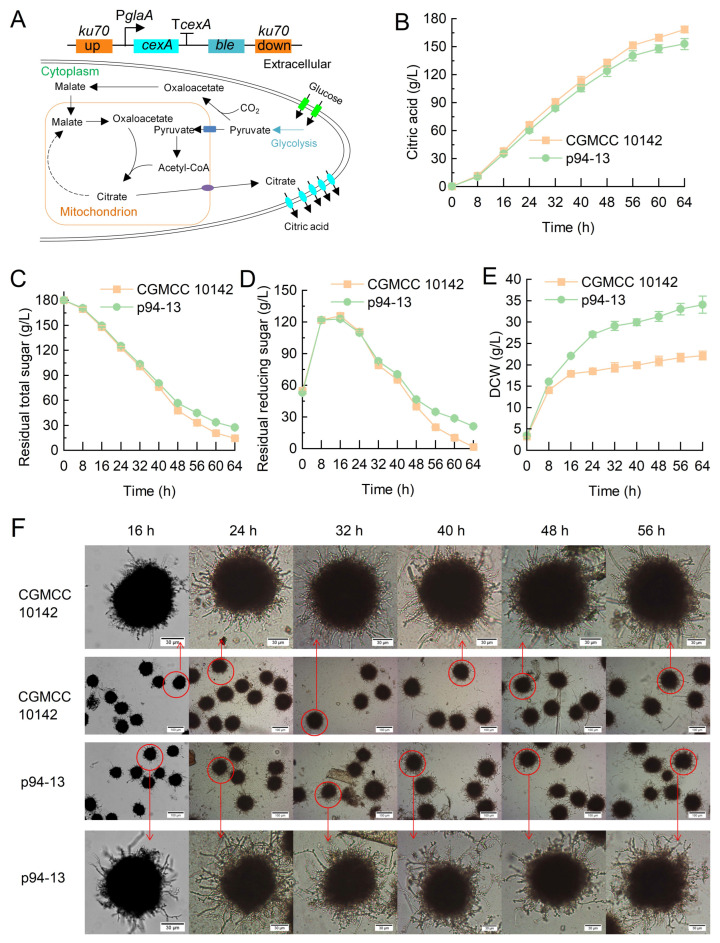
Inductive expression of *cexA* in *A. niger* using the promoter P*glaA*. (**A**) Schematic diagram of *cexA* single overexpression in *A. niger*. (**B**–**E**) Citric acid, residual total sugar, residual reducing sugar, and dry cell weight (DCW) were measured every 8 h in a 30 L fermenter. (**F**) Microscopic observation of the mycelium pellet’s morphology in a 30 L fermenter. The data presented are mean values of at least three independent assays; the error bars represent the standard deviation. Data are given as means ± SD, *n* = 3.

The export of citrate from intracellular to extracellular was also a bottleneck in industry production. Prompt secretion of intracellular citrate into the extracellular space eliminated feedback inhibition caused by citrate accumulation [14]. Therefore, overexpression of *cexA* using the inducible promoter P*glaA* improved citrate secretion under the “pull” effect (Figure 3A).

The dissolved oxygen profiles of CGMCC 10142 and the *cexA*-overexpressing strain p94-13 were nearly identical throughout fermentation (Figure S8C). Both strains reached the minimum DO within 10 h. However, the dissolved oxygen level began to rise gradually during the middle phase of fermentation of p94-13. The pH declined continuously to a final value of 1.5 (Figure S8D). Unsatisfactorily, p94-13 showed no desired improvement in the CA titer. The CA production assay revealed that the final CA titer of p94-13 was 9.2% lower than that of CGMCC 10142, reaching 152.99 g/L (Figure 3B). The apparent sugar-to-acid yield in p94-13 stands at 0.85 g/g, demonstrating a productivity of 2.39 g/h/L. Residual sugar levels displayed that p94-13 consumed sugar at a rate lower than CGMCC 10142 (Figure 3C,D). After fermentation, the residual total sugar of p94-13 reached 27.56 g/L, an increase of 87.48% over CGMCC 10142. At each detection time point, it can be observed that p94-13 formed looser mycelium pellets with elongated hyphae and more apical branching and produced more biomass than CGMCC 10142 (Figure 3E,F). After fermentation, the biomass assay demonstrated that the final DCW of p94-13 was 53.69% higher than that of CGMCC 10142, reaching 34.07 g/L (Figure 3E), illustrating the impact of CexA on the growth and morphology of *A. niger*. Phenotypic analysis revealed that the overexpression of *cexA* caused colony growth defects of *A. niger* (Figure S6). Thus, these findings provide clear evidence that *cexA* expression levels may affect the normal metabolism in *A. niger*.

**Figure 4:**
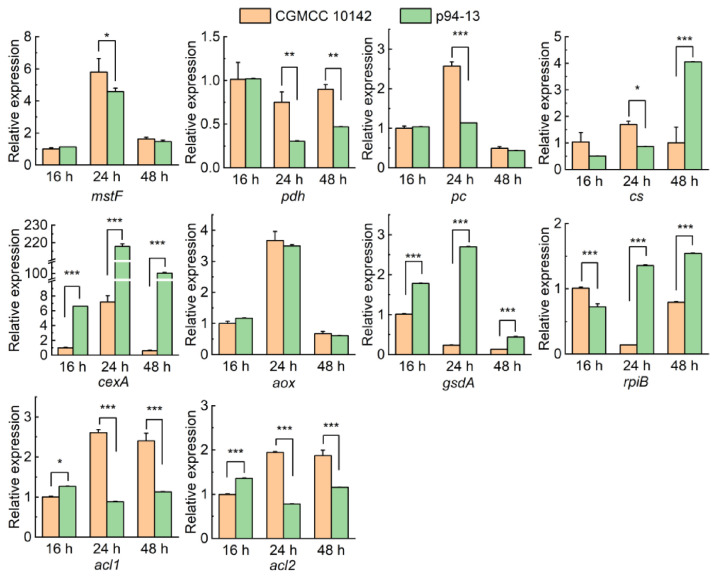
Transcript levels of key genes in p94-13 were assessed at the indicated time points in a 30 L fermenter. The data presented are mean values of at least three independent assays; the error bars represent the standard deviation. Statistical significance was estimated by one-way ANOVA followed by a post hoc Tukey’s multiple comparisons. Asterisks indicate significant differences: * *p* < 0.05; ** *p* < 0.01; *** *p* < 0.001, indicating varying degrees of significance. Data are given as means ± SD, *n* = 3.

Subsequently, transcriptional analysis of key genes further confirmed the effect of CexA on intracellular metabolism. Transcription analyses showed (Figure 4) that the relative expression levels of *mstF*, *pdh*, *pc*, *cs*, *cexA,* and *aox* genes in p94-13 were 0.85-, 0.67-, 0.64-, 1.45-, 36.94-, and 0.98-fold those of CGMCC 10142, respectively. Although the expression level increased, *cs* was consistently expressed at low levels during the critical phase of CA fermentation, which apparently affected the intracellular citrate synthesis rate. Cytoplasmic acetyl coenzyme A (Ac-CoA) is involved in cellular stress, cell membrane synthesis, and histone acetylation [33]. The ATP-citrate lyase (ACL) pathway was the primary cytoplasmic Ac-CoA-generating pathway in *A. niger* [34]. The relative expression levels of *acl1* and *acl2* genes in the p94-13 were 0.54- and 0.68-fold those of CGMCC 10142, respectively. This may lead to a decrease in cytoplasmic Ac-CoA, which caused a decrease in *pc* transcript levels (Figure 4). As observed in p94-13, the relative expression levels of *gsdA* and *rpiB* were 3.58- and 1.86-fold those of CGMCC 10142 (Figure 4), respectively, consistent with the phenomenon of increased biomass (Figure 3E). In conclusion, *cexA* overexpression impaired dense mycelium pellet formation and downregulated key genes involved in CA synthesis, resulting in lower CA production.

### 3.5. Simultaneous Overexpression of mstF and cexA Improves CA Production Efficiency and Fermentation Performance

**Figure 5:**
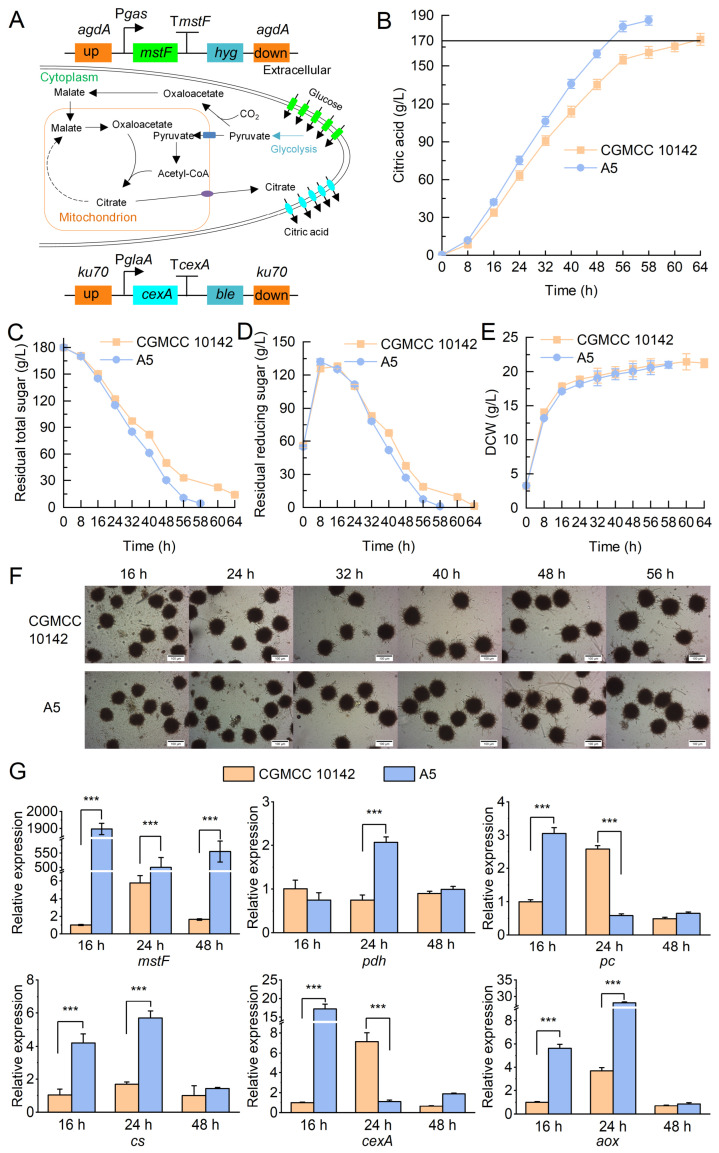
Simultaneous overexpression of *mstF* and *cexA* in *A. niger*. (**A**) Schematic diagram of simultaneous overexpression of *mstF* and *cexA* in *A. niger*. (**B**–**E**) Citric acid, residual total sugar, residual reducing sugar, and dry cell weight (DCW) were measured every 8 h in a 30 L fermenter. (**F**) Microscopic observation of the mycelium pellet’s morphology in a 30 L fermenter. (**G**) Transcript levels of key genes were measured at the indicated timepoints. The data presented are mean values of at least three independent assays; the error bars represent the standard deviation. Statistical significance was estimated by one-way ANOVA followed by a post hoc Tukey’s multiple comparisons. Asterisks indicate significant differences: *** *p* < 0.001, indicating varying degrees of significance. Data are given as means ± SD, *n* = 3.

Enhanced glucose import capacity improved citrate synthesis efficiency and glucose conversion but was limited by the citrate export. In contrast, increasing citrate export capacity by overexpressing *cexA* alone affected mycelium pellet formation, interfered with intracellular metabolism, and reduced CA production. We speculated that the reason may be that the intracellular synthesis of citrate, coupled with its extracellular transport, failed to achieve a dynamic equilibrium. Therefore, we expect to improve the CA fermentation efficiency by simultaneously enhancing glucose import and citrate export under the “push and pull” effect (Figure 5A).

During fermentation, the trend in dissolved oxygen concentrations for A5 and CGMCC 0142 was nearly identical (Figure S8E). The dissolved oxygen level of the *mstF/cexA* co-overexpressing strain A5 declined rapidly in the early stage, reaching 10% at 5 h. The pH value decreased continuously during fermentation, reaching a final value of 1.4 (Figure S8F). The CA production assay revealed that the final CA titer of A5 was 8.71% higher than that of CGMCC 10142, reaching 185.91 g/L (Figure 5B). Crucially, A5 consistently maintained a high fermentation efficiency and completed the fermentation process 6 h earlier than CGMCC 10142 (Figure 5B). Upon calculation, the apparent sugar-to-acid yield in A5 stands at 1.03 g/g, demonstrating a productivity of 3.21 g/h/L. The monitoring of residual sugar levels indicated that A5 consumed sugar at a rate higher than CGMCC 10142 (Figure 5C,D). The total sugar consumption of A5 was 68.41% higher than that of CGMCC 10142, at only 4.47 g/L. Notably, there was no difference in mycelium pellets and biomass (Figure 5E,F) between A5 and CGMCC 10142.

Transcription analyses demonstrated (Figure 5G) that the relative expression levels of *mstF*, *pdh*, *pc*, *cs*, *cexA,* and *aox* genes in A5 were 350.01-, 1.44-, 1.05-, 3.02-, 2.30-, and 6.47-fold those of CGMCC 10142, respectively. Key metabolic enzymes involved in citric acid synthesis were differentially upregulated. Surprisingly, the *mstF* transcript level in A5 was considerably higher than that of H7 (Figure 2G and Figure 5G), whereas the *cexA* transcript level was much lower than that of p94-13 (Figure 4) but higher than that of H7 (Figure 2G). Meanwhile, the high expression of *mstF* had no effect on CA production morphology. High expression of *aox* (Figure 5G) was also observed, which was important for NADH recirculation without ATP production, and uncoupling the inhibitory effect of ATP on glycolysis. In conclusion, simultaneous overexpression of *mstF* and *cexA* in *A. niger* enhanced citrate synthesis flux and export, improving CA production. It is attributed to the dynamic equilibrium between effective substrate import, efficient intracellular citrate synthesis, rapid citrate export, and maintenance of energy balance.

## 4. Discussion

### 4.1. Intracellular Import of Glucose Stands as a Potential Bottleneck in the Synthesis of CA by A. niger

A massive carbon flux through the glycolytic pathway was a fundamental prerequisite to efficient citrate synthesis, as it provided sufficient precursors [20]. And to achieve high efficiency in glycolysis, an efficient glucose transport system was required. Recombinants we constructed demonstrated improvements in terms of fermentation strength and sugar–acid conversion for CA fermentation by *A. niger*. These results underscore that the high-affinity glucose transporter accelerated the glucose import. The upregulation of CS (Figure 2G) contributed to citrate accumulation. However, a trace increase in *cexA* transcripts further limited efficient CA production. Although *mstF* was highly expressed (Figure 2G and Figure 5G), no changes in mycelium pellet morphology were observed, indicating that MstF had no effect on the mycelium pellet morphology formation, in contrast to CexA.

### 4.2. Overexpression of cexA Affects Mycelia Morphogenesis and Transcription of Key Genes

The enhanced glucose import increased citrate synthesis flux and CA accumulation. However, the export of citrate further limited fermentation potential. In this study, P*glaA*-induced *cexA* strain p94-13 formed mycelium pellets with elongated hyphae and more apical branching and showed transcriptional changes in key genes of central metabolism. The degradation of cytosolic citrate constitutes the primary source of cytosolic acetyl-CoA [34], while simultaneously consuming ATP and releasing feedback inhibition. Although CexA provokes a “pull” effect on citrate, it may cause cytoplasmic citrate depletion, thereby affecting other biological processes. The transcriptional downregulation (Figure 4) of key genes *pdh*, *pc*, and *acl* confirmed this hypothesis. During CA fermentation, redox balance was important. AOX was responsible for oxidizing excess NADH without forming ATP, and the lysis of cytoplasmic citrate may help release the pressure of the alternative respiration pathway [20]. But *aox* transcript levels were unchanged (Figure 4). Downregulation of *acl* transcription may enable excess ATP to inhibit glycolysis, redirecting metabolic flux toward the PPP pathway. As we observed, *gsdA* and *rpiB* showed upregulation (Figure 4), and biomass increased (Figure 3E) in p94-13. At the same time, cytoplasmic Ac-CoA was closely related to cell membrane synthesis and the acetylation of nuclear histones [35]. Increased Ac-CoA in the cytoplasm effectively mitigated pH inhibition and promoted cell growth under stress conditions [33,35,36]. On the other hand, changes in mycelium pellet morphology induced by *cexA* overexpression may also affect the strain’s stress tolerance and oxidative stress. NADPH can defend cells from oxidative stress by reacting directly or indirectly with ROS [37]. Metabolic flux was reconfigured in response to oxidative stress, enhancing the PPP flux to increase NADPH supply [38,39,40]. This may be one reason for *gsdA* transcript upregulation and increased biomass. Our findings were consistent with previous studies. Previous studies demonstrated that tet-on induced *cexA* expression yielded higher CA production than the constitutive expression of P*mbfA*, and constitutive expression showed a reduced conidiation pattern [24]. These findings demonstrated that citrate exporter CexA exhibited a diversity of biological functions. And other organic acid transporter proteins exhibited the diversity of biological functions, such as tetracarboxylic acid transporters. In *Metarhizium acridum, MaDct1* not only regulated and transported tetracarboxylic acid but also modified fungal development and conidiation by influencing carbon source availability, and was involved in stress tolerance [41]. This drives the need for metabolic regulation in terms of transporters to shift from static regulation of simple intracellular overexpression to dynamic regulation in response to cell growth and metabolites [42]. In conclusion, a profound comprehension of the structure–function and transcription–regulation relationships of organic acid transporters can facilitate the industrialization of efficient organic acid production.

### 4.3. Dynamic Equilibrium Between Glucose Import and Citrate Export Can Improve the Fermentation Efficiency of CA by A. niger

Overexpressing transporters increases glucose import and citrate export, driving the carbon reaction forward for CA accumulation under the “push–pull” effect. Nevertheless, the simple overexpression of a transporter often does not lead to the expected accumulation of metabolites [43]. In the malic acid fermentation of *Trichoderma reesei*, the titer of malic acid reached 235.8 g/L through the overexpression of the glucose transporter, key enzymes for malic acid synthesis, and malic acid transporter, and optimization of fungal morphology [21]. Research results emphasize that collaboration between transport modules, metabolic modules, and morphological modules contributes to organic acid production.

The engineered strains we developed demonstrated a significant improvement in three key metrics of CA synthesis: increased CA production, reduced fermentation time, and enhanced sugar-acid conversion. These results highlight the vital role of efficient transporters in producing high-quality CA. Simultaneous overexpression of *mstF* and *cexA* provoked “pull” and “push” effects in the metabolic network, increasing intracellular citrate synthesis metabolic flux and export. At the same time, the cyanide-resistant respiration (CRR) pathway was enhanced. Transcript levels of *aox* were up-regulated (Figure 5G), leading to NADH recirculation and uncoupling the inhibition of ATP production on the glycolytic pathway and CS. To gain insight into the effect of AOX on energy metabolism in *A. niger* CGMCC 10142, we have previously conducted *aox* disruption and overexpression strains [7]. Overexpression of *aox* increased the NADH oxidation rate, thereby accelerating the rate-limiting step and enhancing the metabolic flux. Furthermore, AOX critically contributed to maintaining mycelium pellet morphology under oxidative stress. Of course, the mechanism of the CRR pathway enhancement caused by *mstf*/*cexA* co-overexpression remains unclear. Numerous cases underscore the important role of NADH recycling in the synthesis of organic acids [44,45]. These results illustrate the importance of maintaining energy balance in metabolic regulatory strategies to enhance organic acids.

Based on the above analysis, the systematic metabolic regulation of organic acid synthesis in filamentous fungi needs to consider the following key points: (1) increasing the intracellular import of carbon sources; (2) improving the intracellular synthesis flux of organic acids; (3) enhancing the extracellular export of organic acids; (4) accelerating the NADH recirculation and decoupling the inhibitory effect of ATP; and (5) maintaining the optimal mycelium pellet morphology for organic acids synthesis. Unilateral efforts may not achieve the desired results; a balance will need to be struck between these several strategies. This requires shifting from the “push–pull-block” strategy of regulating enzyme expression under static control to precise dynamic regulation [42]. Modular engineering becomes a promising strategy [46]. Modular design of independent and functionally specific components, such as the morphology module, transporter module, and metabolism module, and adjustment of the relative activity of the modules can precisely regulate the metabolic synthesis of organic acids. The dynamic control system can regulate the balance between intracellular functional modules by responding to changes in signals to maximize the carbon flux to synthesize the target metabolite. The P*mfsA* promoter sensed calcium ions to trigger the expression of target genes in *A. niger* malic acid fermentation [47]. Precise control of *sthA* expression by the P*mfsA* promoter during the fermentation phase mitigated the severe asexual developmental defects caused by constitutive expression of *sthA*. Therefore, it is essential to tap into signal-sensing key metabolic regulation factors to control gene expression precisely.

## 5. Conclusions

In summary, this study underscored the key role of the transporter in CA biosynthesis. Overexpression of the *mstF* gene increased the apparent sugar-to-acid yield and metabolic flux of citrate synthase but had no effect on CA production morphology. In addition, the overexpression of *cexA* impaired CA production and mycelium pellet formation. Simultaneous overexpression of *mstF* and *cexA* achieved a dynamic equilibrium between key modules of citrate synthesis, increasing CA accumulation and fermentation efficiency. The ultimate co-overexpression engineered strain A5 exhibited exceptional performance in CA synthesis within a 30 L fermenter, showing elevated CA titer (185.91 g/L), apparent sugar-to-acid yield (1.03 g/g sugar), productivity (3.21 g/h/L), and a shortened fermentation cycle (from 64 to 58 h).

## Figures and Tables

**Table 1:** Microbial cell factories for producing citric acid.

Microorganism	Substrate	Cycle (h)	Scale	Citric Acid (g/L)	Reference
*A. niger* TNA 101	Liquefied corn medium, 180 g/L total sugar	72	30 L	185.7	[2]
*A. niger* YX-1217	Liquefied corn medium, 175.2 g/L initial reducing sugar	60	250 mL	180.0	[3]
*A. niger*	Liquefied corn medium, 180 g/L total sugar	58	5000 L	181.4	[4]
*A. niger*	Liquefied corn medium, 170 g/L total sugar	54	5000 L	171.8	[4]
*A. niger*	Liquefied corn medium, 200 g/L total sugar	69	5000 L	193.4	[4]
*A. niger*	corn meal hydrolysate, 210 g/L total sugar	60	50 L	187.5	[11]
*A. niger*	Liquefied corn medium, 175 g/L total sugar	55	24 L	173.2	[12]
*A. niger chsC-3*	Liquefied corn medium, 177 g/L total sugar	64	30 L	180.3	[13]

## Data Availability

All data supporting the findings of this study are available within the paper and its Supplementary Information.

## Supplementary Materials

The following supporting information can be downloaded at https://www.mdpi.com/article/10.3390/jof12070472/s1. Table S1: Strains used in the study. Table S2: Plasmids used in the study. Table S3: Primers used in this study. Table S4: The screening of high-yield citric acid transformants with single *mstF* overexpression in CM medium with low glucose (0.05%) containing 0.2% CaCO3. Table S5: The screening of high-yield citric acid transformants with simultaneous overexpression of *mstF* and *cexA* in CM medium with low glucose (0.05%) containing 0.2% CaCO3. Figure S1: Schematic Diagram of plasmid construction. Figure S2: PCR verification of *mstF* overexpression recombinants. Figure S3: PCR verification of the *cexA* overexpression recombinants. Figure S4: PCR verification of simultaneous overexpression *cexA* and *mstF* recombinants. Figure S5: Microscopic observation of mycelium pellets morphology under shake flask fermentation. Figure S6: Colony diameter of A. niger p94-13 and CGMCC 10142 on PDA plate. Figure S7: Validating *cexA* expression cassette and/or *mstF* expression cassette in p94-13, H7, A5 at the 15th generation. Figure S8: Dissolved oxygen and pH profiles of strains H7 (*mstF* overexpression), p94-13 (*cexA* overexpression), A5 (*mstF*/*cexA* co-overexpression), and control strain CGMCC 10142 during 30 L fed-batch fermentation.

## Abbreviations

MstF, high-affinity glucose transporter; CexA, citrate exporter; CA, citric acid; PDH, pyruvate dehydrogenase; PC, pyruvate carboxylase; CS, citrate synthase; AOX, alternative oxidase; GsdA, glucose 6-phosphate dehydrogenase; RpiB, ribose 5-phosphate isomerase; ACL, ATP-citrate lyase; CRR, cyanide-resistant respiration; and PPP, pentose phosphate pathway.
