# Supplementary material for: Transporter Engineering for Enhancing Citric Acid Production in Aspergillus niger
Source: J Fungi (Basel). 2026 Jun 26;12(7):472. doi: 10.3390/jof12070472 (PMC13413353; doi:10.3390/jof12070472)
Supplement: Supplementary file 1 [file jof-12-00472-s001.zip › jof-4342615-supplementary.pdf]

Transporter engineering for enhancing citric acid production in *Aspergillus niger*

Jie Li[1], Mingyang Li[1], Yan Song[1], Zeyu Xu1, Yue Chen[1], Xianli Xue[1*], Depei Wang[1,2,3*]

[*] **Corresponding author:**

Xianli Xue (E-mail: xuexianli@tust.edu.cn)

Depei Wang (E-mail: wangdp@tust.edu.cn)

Authors:

Jie Li (E-mail: joylee6863@163.com)

Mingyang Li (E-mail: limingyang@mail.tust.edu.cn)

Yan Song (E-mail: songyansy@mail.tust.edu.cn)

Zeyu Xu (E-mail: xuzy@mail.tust.edu.cn)

Yue Chen (E-mail: yuechen@tust.edu.cn)

[1] Key Laboratory of Industrial Microbiology & Engineering Research Center of Food Biotechnology of Ministry of Education, College of Biotechnology, Tianjin University of Science and Technology, Tianjin 300457, China.

[2] Key Laboratory of Industrial Fermentation Microbiology (Tianjin University of Science and Technology), Ministry of Education, Tianjin 300457, China.

[3] Tianjin Engineering Research Center of Microbial Metabolism and Fermentation Process Control, College of Biotechnology, Tianjin University of Science and Technology, Tianjin 300457, China.

Table S1. Strains used in the study.

Strain	Relevant genotype/description	Source
A. niger CGMCC 10142	Citric acid producer	Tianjin University of Science & Technology
A. niger H7	P*gas-mstF*, *hyg*^{R}	This study
A. niger p94-13	P*glaA-cexA*, *ble*^{R}	This study
A. niger A5	P*gas-mstF*, *hyg*^{R}; P*glaA-cexA*， *ble*^{R}	This study

Table S2. Plasmids used in the study.

Plasmid	Description	Source
p44	PCR template for amplifying *hyg* and vector backbone	Tianjin University of Science & Technology
p60	PCR template for amplifying *ble*	Tianjin University of Science & Technology
p94	P*glaA*:*cexA*:T*cexA*, *ble*^{R}, *cexA* overexpression	This study
p95	P*gas*:*mstf*:T*mstF*, *hyg*^{R}, *mstF* overexpression	This study

Table S3. Primers used in this study.

Name	Sequence(5'-3')	Intention
plasmid construction primers		
P94ku70L-F	ctatgacatgattacgaattcGAGGCCAAACAGGCAGACAC	for amplifying *ku70* upstream sequence
P94ku70L-R	aatggcaATCGCTGGGCAATGACTTGT	
P94Pgla-F	attgcccagcgatTGCCATTGGCGGAGGGGT	for amplifying *gla* promoter
P94Pgla-R	ggttgaagacatTGCTGAGGTGTAATGATGCTGG	
P94cexA-F	cctcagcaATGTCTTCAACCACGTCTTCATCA	for amplifying the ORF and terminator of *cexA*
P94cexA-R	ttctgtcgacCAAACAACAGAACTTCTTGCTTTATACT	
P94ble-F	ctgttgtttgGTCGACAGAAGATGATATTGAAGGAG	for amplifying *ble*
P94ble-R	ttcggcagctgcTCAGTCCTGCTCCTCGGCC	
P94ku70R-F	aggactgaGCAGCTGCCGAAGGGAGT	for amplifying *ku70*

P94ku70R-R	acgacggccagtgccaagcttTAACTGTACATCGCCTAAAATACGTG	downstream sequence
P95agdAL-F	tatgaccatgattacgaattcATGGTGAAGTTGACGCATCTCC	for amplifying *agdA* upstream sequence
P95agdAL-R	aatataataaCCGGGGCGGGGCCTGAGT	
P95Pgas-F	cccgccccggTTATTATATTGTGATTATTTTTATTCTGGGTG	for amplifying *gas* promoter
P95Pgas-R	ccaatcaacatGTGAGGAGGTGAACGAAAGAAGA	
P95MstF-F	cctcctcacATGTTGATTGGCAACATCTACGTG	for amplifying the ORF and terminator of *mstF*
P95MstF-R	acgtcgacTACTTTGATTGCTTTGTCGTCTATTTT	
P95HYG-F	gcaatcaaagtaGTCGACGTTAACTGATATTGAAGGAG	for amplifying *hyg*
P95HYG-R	caaccgcagccaatgttCTATTTCTTTGCCCTCGGACG	
P95agdAR-F	gaaatagAACATTGGCTGCGGTTGAGA	for amplifying *agdA* downstream sequence
P95agdAR-R	acgacggccagtgccaagcttTAGATGGGATTTAAAGTCTTCACTCATG	
RT-qPCR primers		
mstA-F	TGGCTCCTACCGTATCCCAA	*mstA* transcription level analysis
mstA-R	CAAGAACCTCCGCAGCCTTA	
mstE-F	AGGCCGCAAGATGAACTCAA	*mstE* transcription level analysis
mstE-R	ACGTGAGCAGAGGGATAGGT	
mstF-F	ATCTACGTGATTGCGAGCGT	*mstF* transcription level analysis
mstF-R	AGGGCCCTGGTTGAAATAGC	
mstG-F	TGTACGGTGTGCCTGAGAAC	*mstG* transcription level analysis
mstG-R	GCCCATACCCAGGCAGATAC	
mstH-F	TGTCGGAATGAGTGTGCTCC	*mstH* transcription level analysis
mstH-R	CCCACGCTCATCTCGTACTC	
pdh-F	CGTTCTGTTCGGTTGTGAGA	*pdh* transcription level analysis
pdh-R	GCCGTTGACCTTGATACCA	
pc-F	TTACTCCTCCCTCCCTGACA	*pc* transcription level analysis
pc-R	TGGACAGCCTTGATACCGA	
cs-F	TCAGATTGAAGTGCTTTCGCTA	*cs* transcription level analysis
cs-R	CATCTTGGTGAGCCAGTT	
cexA-F	CCCTGGACATTGGACTCACT	*cexA* transcription level analysis
cexA-R	TCGGTAAGACGGTGGTTGTA	
aox-F	AGCTTGGGTCCATCCTGTCT	*aox* transcription level analysis
aox-R	AGTCACAAGATCCATGCCCC	
gsdA-F	CGTTGCCCGTATCATCGTA	*gsdA* transcription level analysis
gsdA-R	CCTCTTCCTTCCAGTTAGGCTC	
rpiB-F	ACAAGGAGACCCTGAAGGCT	*rpiB* transcription level analysis
rpiB-R	GCAGATGAAGAGACCACGGT	
acl1-F	GTGTCAAGTCCCGCAACAAC	*acl1* transcription level analysis
acl1-R	GGTCACAGTCTCGACAGCAA	
acl2-F	GAACGAGCACAAGGTCCAGA	*acl2* transcription level analysis
acl2-R	ATGTTGAGGCCAAGCTCCTC	
actA-F	ACCACCGACTCCCTACTA	*actA* transcription

actA-R	AGTCAAGAGAGAGAGATGGGAT	level analysis

Table S4. The screening of high-yield citric acid transformants with single *mstF* overexpression in CM medium with low glucose (0.05%) containing 0.2% $CaCO_3$.

Strains	Colony diameter / cm	Acid ring diameter / cm	Ring diameter ratio	Strains	Colony diameter / cm	Acid ring diameter / cm	Ring diameter ratio
CGMCC 10142	0.85	1.93	2.26	p95 H9	0.75	1.8	2.4
p95 H1	0.85	2.25	2.65	p95 H10	0.98	2.28	2.33
p95 H2	0.93	1.85	2	p95 H11	0.9	2.1	2.33
p95 H3	0.83	1.78	2.15	p95 H12	0.73	1.68	2.31
p95 H4	1	2.48	2.48	p95 H13	0.95	2.18	2.29
p95 H5	0.75	1.83	2.43	p95 H14	1.15	2.53	2.2
p95 H6	0.8	1.98	2.47	p95 H15	1.2	2.6	2.17
p95 H7	0.95	2.45	2.58	p95 H16	1.1	2.28	2.07
p95 H8	0.85	2.05	2.41	p95 H17	0.9	2.1	2.33

Table S5. The screening of high-yield citric acid transformants with simultaneous overexpression of *mstF* and *cexA* in CM medium with low glucose (0.05%) containing 0.2% $CaCO_3$.

Strains	Colony diameter / cm	Acid ring diameter / cm	Ring diameter ratio	Strains	Colony diameter / cm	Acid ring diameter / cm	Ring diameter ratio
CGMCC 10142	0.85	1.93	2.26	A10	1.03	2.15	2.1
A1	0.88	2.43	2.77	A11	0.7	1.95	2.79
A2	0.8	2.2	2.75	A12	0.98	2.25	2.31
A3	0.9	2.3	2.56	A13	0.7	1.9	2.71
A4	0.9	2.45	2.72	A14	0.93	2.45	2.65
A5	0.75	2.15	2.87	A15	0.85	1.95	2.29
A6	0.83	2.15	2.61	A16	0.95	2.25	2.37
A7	0.73	2.1	2.90	A17	0.93	2.2	2.38
A8	0.83	2.35	2.85	A18	0.85	2.08	2.44
A9	1	2.28	2.28	A19	0.9	2.38	2.64

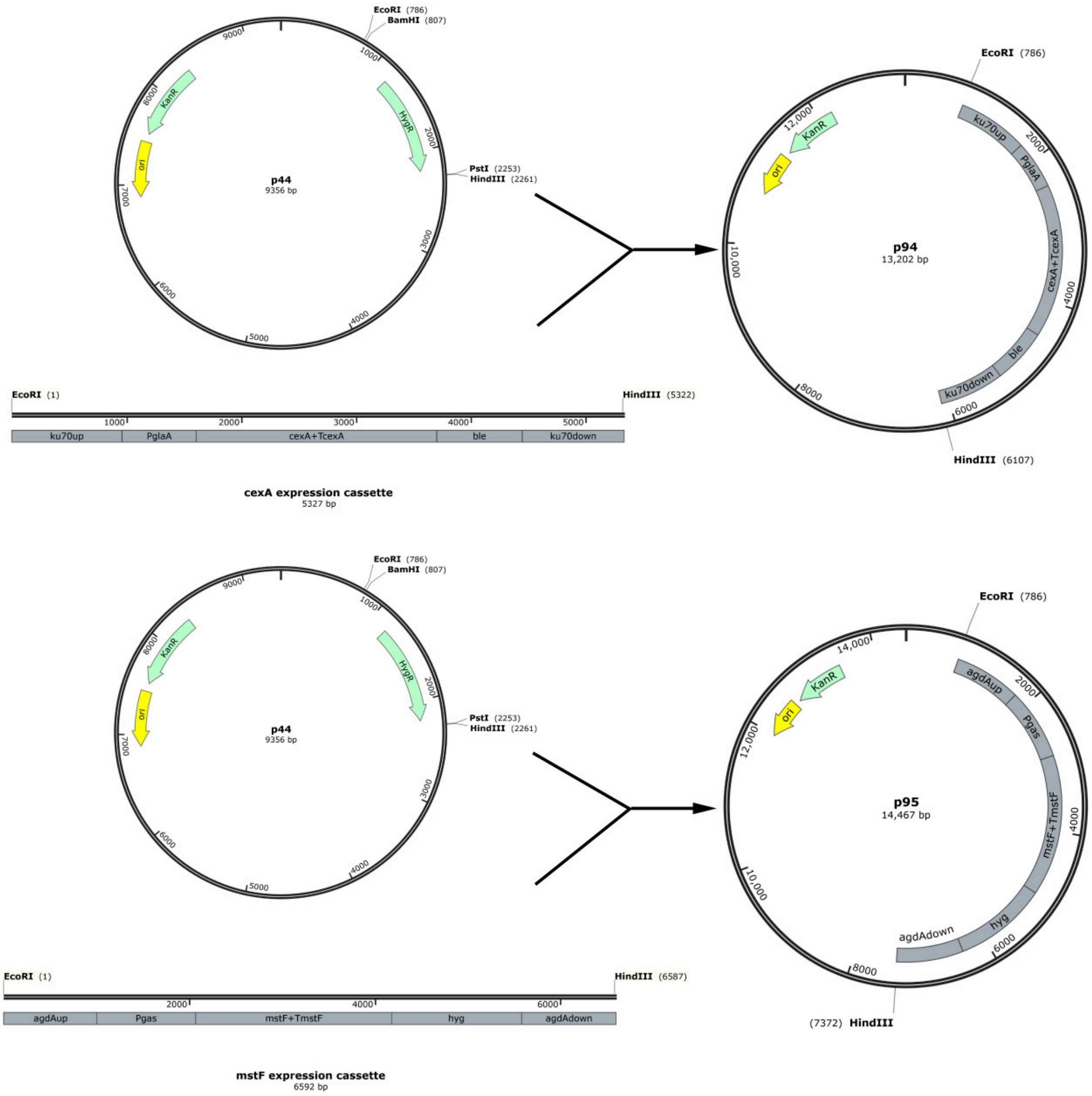

Figure S1. Schematic Diagram of plasmid construction.

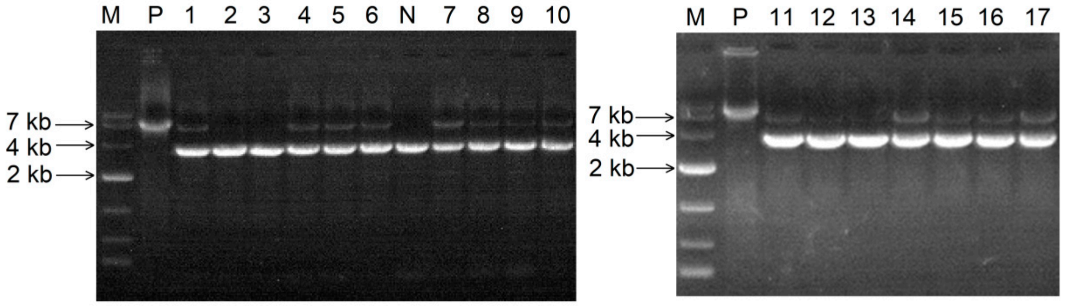

Figure S2. PCR verification of *mstF* overexpression recombinants. M: DL10000 DNA Marker. P: The p95 plasmid was used to amplify the expression frame full length as a template positive control. N:

The genome of *A. niger* CGMCC 10142 was used as a template negative control. Lane 1-17: The genome of recombinants was used as a template. 1-17 represent the recombinants p95 H1-H17, respectively. Primers: P95agdAL-F/P95agdAR-R. The integration location was determined using primers spanning the total length of *agdA*. If the *mstF* expression cassette was homologously recombined to the *agdA* gene, a 6950 bp fragment could only be amplified by PCR. If the *mstF* expression cassette was not integrated into the genome, a 3499 bp fragment could only be amplified by PCR. If the *mstF* expression cassette was randomly integrated the genome, fragments of 3499 bp and 6950 bp could be amplified by PCR. The results showed that two bands can be amplified from the transformants (H1, H3-H11, H14-H17). A band of 6590 bp was consistent with the band amplified *mstF* expression cassette from the p95, and another band of 3499 bp was consistent with the band amplified *agdA* from the genomic DNA of the CGMCC 10142, indicating that *mstF* was overexpressed in *A.niger* through random integration.

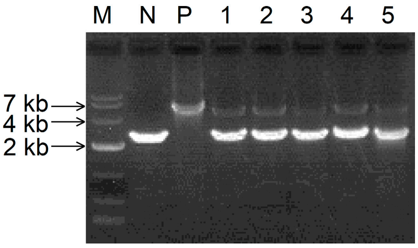

Figure S3. PCR verification of the *cexA* overexpression recombinants. M: DL10000 DNA Marker. N: The genome of *A. niger* CGMCC 10142 was used as a template negative control. P: The p94 plasmid was used to amplify the expression frame full length as a template positive control. Lane 1-5: The genome of recombinants was used as a template. 1-5 represent the recombinants p94-2, p94-13, p94-79, p94-81, and p94-82, respectively. Primers: P94ku70L-F/P94ku70R-R. The integration location was determined using primers spanning the total length of *ku70*. If the *cexA* expression cassette was

homologously recombined to the *ku70* gene, a 5327 bp fragment could only be amplified by PCR. If the *cexA* expression cassette was not integrated into the genome, a 2600 bp fragment could only be amplified by PCR. If the *cexA* expression cassette was randomly integrated the genome, fragments of 2600 bp and 5327 bp could be amplified by PCR. The results showed that two bands can be amplified from the transformants. A band of 5327 bp was consistent with the band amplified *cexA* expression cassette from the p94, and another band of 2600 bp was consistent with the band amplified *ku70* from the genomic DNA of the CGMCC 10142, indicating that *cexA* was overexpressed in *A.niger* through random integration.

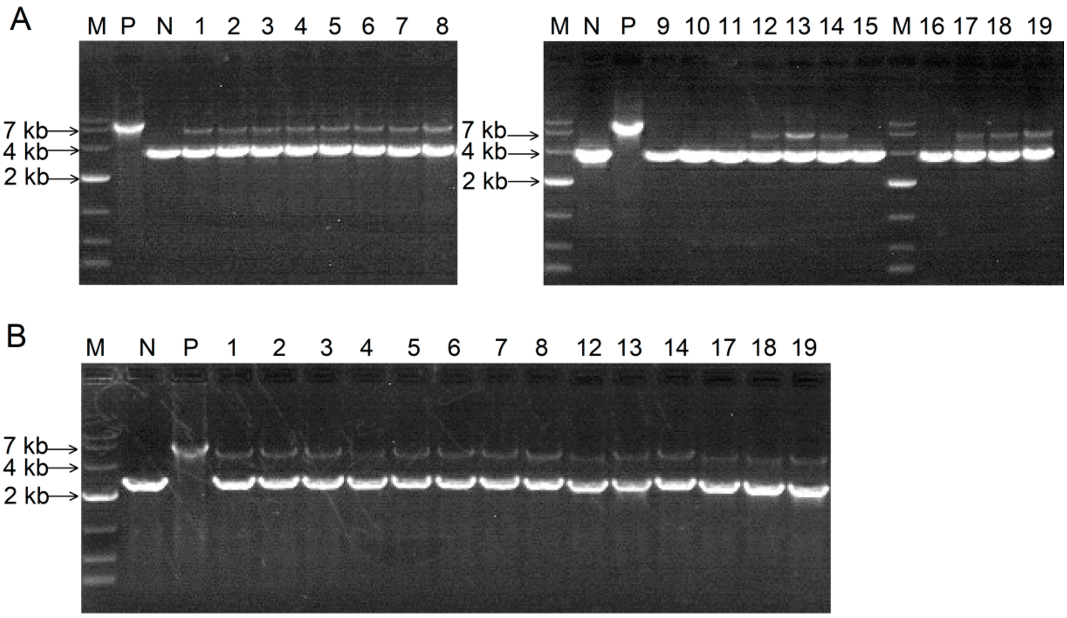

Figure S4. PCR verification of simultaneous overexpression *cexA* and *mstF* recombinants. (A) PCR verification of the *mstF* expression cassette. M: DL10000 DNA Marker. P: The p95 plasmid was used to amplify the expression frame full length as a template positive control. N: The genome of *A. niger* CGMCC 10142 was used as a template negative control. Lane 1-19: The genome of recombinant strain was used as a template to amplify the *mstF* expression frame full length. Primers: P95agdAL-F/P95agdAR-R. 1-19 represent the recombinants A1-A19, respectively. The results showed

that two bands of 3499 bp and 6950 bp can be amplified from the transformants (A1-A8, A12-A14, A17-A19). (B) PCR verification of the *cexA* expression cassette. M: DL10000 DNA Marker. N: The genome of *A. niger* CGMCC 10142 was used as a template negative control. P, The p94 plasmid was used to amplify the expression frame full length as a template positive control. Lane 1-19, The genome of recombinants was used as a template to amplify the *cexA* expression frame full length. Primers: P94ku70L-F/P94ku70R-R. The results showed that two bands of 2600 bp and 5327 bp can be amplified from the transformants (A1-A8, A12-A14, A17-A19). These results indicated that *mstF* and *cexA* were simultaneously overexpressed in *A.niger* through random integration.

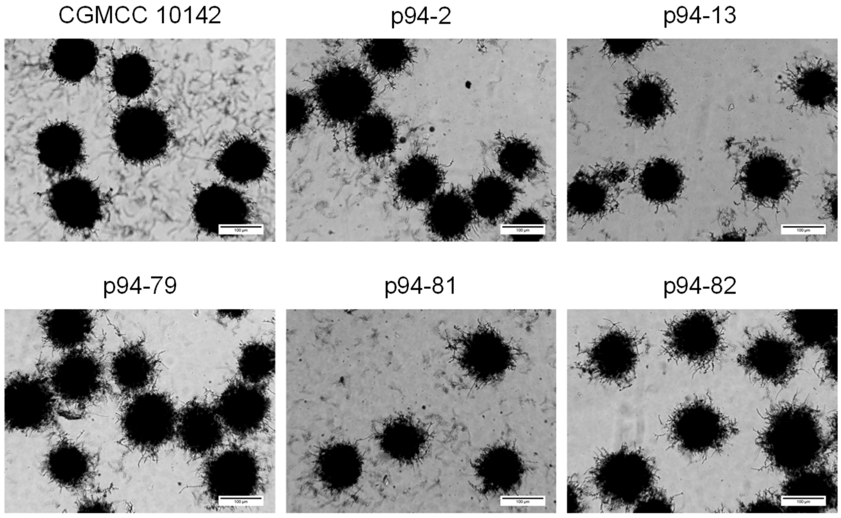

Figure S5. Microscopic observation of mycelium pellets morphology under shake flask fermentation.

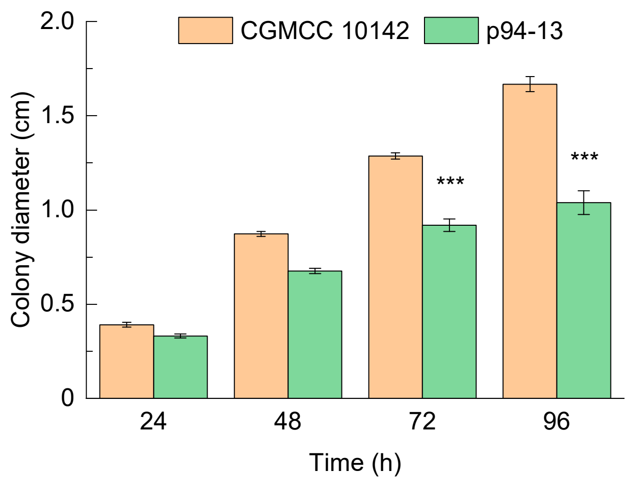

Figure S6. Colony diameter of *A. niger* p94-13 and CGMCC 10142 on PDA plate. *** represents a p < 0.001.

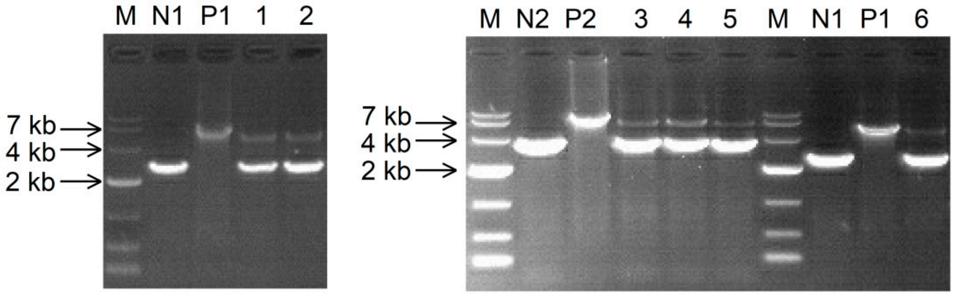

Figure S7. Validating *cexA* expression cassette and/or *mstF* expression cassette in p94-13, H7, A5 at the 15th generation. M: DL10000 DNA Marker. N1, N2: The genome of *A. niger* CGMCC 10142 was used as a template negative control. P1, The p94 plasmid was used to amplify the *cexA* expression cassette as a template positive control. P2: The p95 plasmid was used to amplify the *mstF* expression cassette as a template positive control. Lane 1, 2: The genome of p94-13 was used as a template to amplify the *cexA* expression cassette. Lane 3: The genome of H7 was used as a template to amplify the *mstF* expression cassette. Lane 4, 5: The genome of A5 was used as a template to amplify the *mstF* expression cassette. Lane 6: The genome of A5 was used as a template to amplify the *cexA* expression cassette.

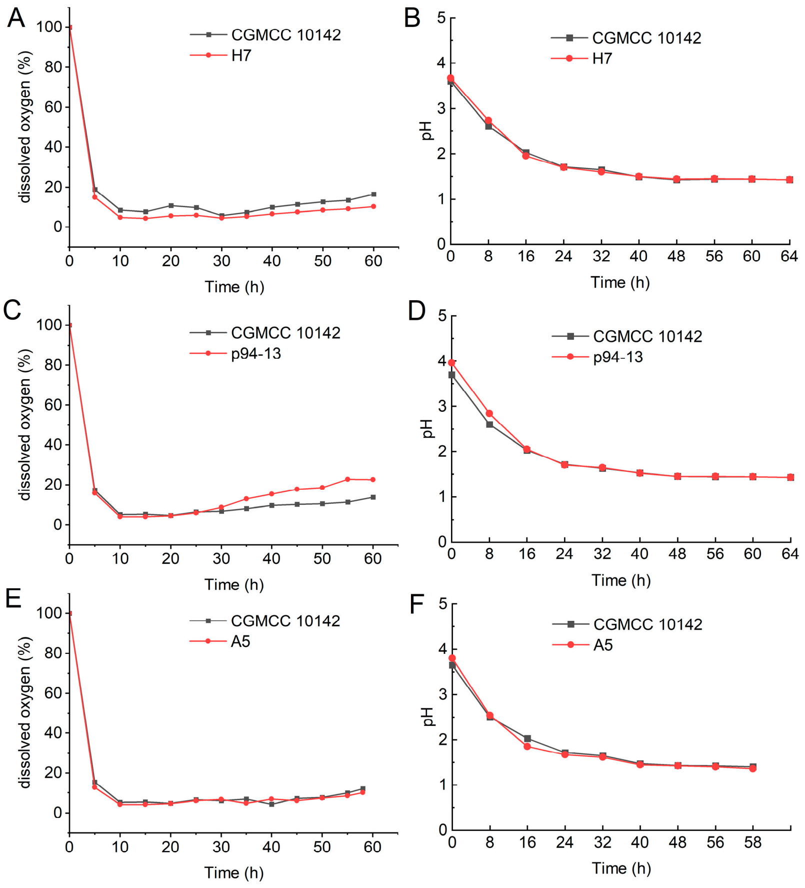

Figure S8 Dissolved oxygen and pH profiles of strains H7 (*mstF* overexpression)，p94-13 (*cexA* overexpression), A5 (*mstF/cexA* co-overexpression), and control strain CGMCC 10142 during 30 L fed-batch fermentation. Panels (A, C, E) show the time-course changes in dissolved oxygen (%) throughout the fermentation period. Panels (B, D, F) display the corresponding pH profiles.

(A, B) *mstF*-overexpressing strain H7 and the control strain CGMCC 10142;

(C, D) *cexA*-overexpressing strain p94-13 and the control strain CGMCC 10142;

(E, F) *mstF/cexA* co-overexpressing strain A5 and the control strain CGMCC 10142.
